# Exome Sequencing Analysis Identifies Compound Heterozygous Mutation in *ABCA4* in a Chinese Family with Stargardt Disease

**DOI:** 10.1371/journal.pone.0091962

**Published:** 2014-03-14

**Authors:** Yu Zhou, Siyu Tao, Hui Chen, Lulin Huang, Xiong Zhu, Youping Li, Zhili Wang, He Lin, Fang Hao, Zhenglin Yang, Liya Wang, Xianjun Zhu

**Affiliations:** 1 Sichuan Provincial Key Laboratory for Human Disease Gene Study and Institute of Laboratory Medicine, Sichuan Academy of Medical Sciences and Sichuan Provincial People's Hospital, Chengdu, Sichuan, China; 2 School of Medicine, University of Electronic Science and Technology of China, Chengdu, Sichuan, China; 3 Henan Eye Hospital and Henan Eye Institute, People’s Hospital of Zhengzhou University, Zhengzhou, Henan, China; 4 Department of Ophthalmology, Sichuan Academy of Medical Sciences and Sichuan Provincial People’s Hospital, Chengdu, Sichuan, China; 5 Laboratory Animal Institute, Sichuan Academy of Medical Sciences and Sichuan Provincial People’s Hospital, Chengdu, Sichuan, China; 6 Chengdu Institute of Biology, Chinese Academy of Sciences and Chinese Academy of Sciences Sichuan Translational Medicine Research Hospital, Chengdu, Sichuan, China; 7 Key Laboratory for NeuroInformation of Ministry of Education, University of Electronic Science and Technology of China, Chengdu, Sichuan, China; 8 College of Life Sciences and Engineering, Xinan Jiaotong University, Chengdu, Sichuan, China; University of Iowa, United States of America

## Abstract

Stargardt disease is the most common cause of juvenile macular dystrophy. Five subjects from a two-generation Chinese family with Stargardt disease are reported in this study. All family members underwent complete ophthalmologic examinations. Patients of the family initiated the disease during childhood, developing progressively impaired central vision and bilateral atrophic macular lesions in the retinal pigmental epithelium (RPE) that resembled a “beaten-bronze” appearance. Peripheral venous blood was obtained from all patients and their family members for genetic analysis. Exome sequencing was used to analyze the exome of two patients II1, II2. A total of 50709 variations shared by the two patients were subjected to several filtering steps against existing variation databases. Identified variations were verified in all family members by PCR and Sanger sequencing. Compound heterozygous variants p.Y808X and p.G607R of the ATP-binding cassette, sub-family A (ABC1), member 4 (*ABCA4*) gene, which encodes the ABCA4 protein, a member of the ATP-binding cassette (ABC) transport superfamily, were identified as causative mutations for Stargardt disease of this family. Our findings provide one novel *ABCA4* mutation in Chinese patients with Stargardt disease.

## Introduction

Stargardt disease (STGD), which is also known as juvenile macular degeneration, was first reported by Karl Stargardt in 1909. It is one of the most common hereditary retinal dystrophies with an estimated prevalence of at least 1:10,000 [1], [2], [3]. It presents with a progressive and significant loss in central vision in the first or second decade of life. However, fundus examination is frequently normal early in the course of disease, even when patients already complain of vision loss. At this stage, the clinical diagnosis of Stargardt disease may be missed. Later on, typical fundus manifestations arise, including pigment mottling, frank macular atrophy, a bull’s eye maculopathy and fundus flecks in the macular and the perimacular region [4]. However, it should be noted that Stargardt disease presents with highly variable phenotypes. Histologically, Stargardt disease is associated with significant loss in photoreceptor cells and a massive deposition of lipofuscin-like material in the retinal pigment epithelium, which has also been observed in aging human eyes [6], [7].

STGD is predominantly inherited as an autosomal recessive trait, although an autosomal dominant form has been also described [5]. Both sexes are equally affected. The STGD gene has been mapped to the short arm of chromosome 1 [8] in a narrow genetic interval, subsequently assigned to band p22.1 [9], now known as ATP-binding cassette, sub-family A (ABC1), member 4 (*ABCA4*) [10], [11]. The gene for an autosomal dominant disorder with a similar phenotype has been reported on chromosome 6 [12]. Autosomal dominant families linked to this locus are at least 50 times less common than families that are consistent with autosomal recessive inheritance [13], [14]. Recessively inherited Stargardt disease is likely to be monogenic. Rare cases of STGD or “Stargardt-like” disease phenotypes have been reported with mutations in *PRPH2* [15], [16], *VMD2* [17], *ELOVL4* [5], [18], [19] and *PROM1* [20]. These genes, as well as *ABCA4*, are also associated with clinically distinct phenotypes including retinitis pigmentosa, cone/rod dystrophy and pattern dystrophy.

Known candidate genes for Stargardt disease such as “*ABCA4*” contain many exons and there are hundreds of identified mutations. The cost and time requirement for mutation screening of all coding exons by Sanger sequencing would equal or exceed that of high-throughput next generation sequencing (NGS) analysis. In this study, we applied next generation sequencing technology to identify the disease-causing gene in this family as part of a large cohort study for retinal diseases. Next-generation sequencing, in particular whole-exome sequencing (WES) can now be performed rapidly and at minimal cost, allowing analysis of the coding regions (exome) of the human genome in single individuals or small families, including patients in whom a clear genotype-phenotype correlation is absent or for clinically and genetically heterogeneous conditions [21], [22], [23].

In the present study, disease-associated mutations were identified by WES of the two affected siblings followed by validation in the family affected by Stargardt disease. Our results identified two compound heterozygous disease-segregating mutations, c.C2424G, p.Y808X and c.G1819A, p.G607R, in the *ABCA4* gene. To exclude the possibility that these mutations were polymorphisms, DNA samples of 1000 unaffected individuals were also screened for these mutations.

## Materials and Methods

### Subjects and Clinical Assessment

Study approval was obtained from the Institutional Review Boards of Sichuan Academy of Medical Sciences and Sichuan Provincial People’s Hospital and Henan Eye Hospital and Henan Eye Institute, People’s Hospital of Zhengzhou University. Written informed consent was obtained in accordance with the Declaration of Helsinki for all subjects enrolled in this study. For minors, written consent was obtained from the father. Five family members were evaluated by a retina specialist and underwent complete ophthalmological assessment that included visual acuity measurement, fundus photography, fundus fluorescein angiography (FFA), multifocal electroretinogram (mfERG), optical-coherence tomography (OCT) and computerized visual field testing. In the 1000 normal matched controls, all individuals underwent an eye examination and no signs of eye diseases were observed. Venous blood samples were obtained from all subjects in EDTA Vacutainers.

### DNA Extraction

All genomic DNA was extracted from peripheral blood using a blood DNA extraction kit according to the protocol provided by the manufacturer (TianGen, Beijing, China). DNA samples were stored at −20°C until used. DNA integrity was evaluated by 1% agarose gel electrophoresis.

### Exome Sequencing

Exome sequencing was employed in this study to identify the disease-associated genes. Exome sequencing was performed on DNA samples of the two patients (family members II1 and II2) by Axeq Technology Inc., Seoul, Korea. Each sequenced sample was prepared according to the Illumina protocols. Briefly, one microgram of genomic DNA was fragmented by nebulization, the fragmented DNA was repaired, an 'A' was ligated to the 3' end, Illumina adapters were then ligated to the fragments, and the sample was size selected aiming for a 350−400 base pair product. The size-selected product was PCR amplified, and the final product was validated using the Agilent Bioanalyzer. Streptavidin beads were used to capture probes containing the targeted regions of interest; non-specific binding was then washed out. Then, the sequencing libraries were enriched for the desired target using the Illumina Exome Enrichment protocol and the enriched library validation for quality control analysis were performed by the Axeq Technology. For clustering and sequencing, genomic DNA Illumina TruSeq Exome Capture System (62 Mb) was used to collect the protein coding regions of human genome DNA. It covered 20794 genes and 201121 exons in the Consensus Coding Sequence Region database, approximately 97.2% of CCDS exons or 96.4% of RefSeq exons were captured. (http://www.illumina.com/applications/sequencing/targeted resequencing.ilmn) Each captured library was then loaded onto the Illumina Hiseq2000 sequencer, and we performed high-throughput sequencing for each captured library to ensure that each sample met the desired average sequencing depth. Raw image files were processed by Illumina base calling Software 1.7 for base calling with default parameters and the sequences of each individual were generated as 90-bp pair-end reads.

### Reads, Mapping, and Variant Detection

The high-quality sequencing reads were aligned to the human reference genome (NCBI build 37.1/ hg19) with SOAPaligner (soap2.21). Based on the SOAP alignment results, SOAPsnp v1.05 was used to assemble the consensus sequence and call genotypes in target regions. Data were provided as lists of sequence variants (SNPs and short Indels). For SNP quality control, we filtered SOAPsnp results as follows: (i) Base quality is more than 20; (ii) Depth is between 4 and 200; (iii) Estimate copy number is equal or less than 2; (iv) The distance between two SNPs must be longer than 4. Small Indel detection was performed using the UnifiedGenotyper tool from GATK (version v1.0.4705) after all the high-quality reads were aligned to the human reference genome using BWA (version 0.5.9-r16). SNP and Indel detection were performed only on the targeted exome regions and flanking regions within 200 bp.

### Filtering and Annotation

The detected variants were annotated and filtered based on four databases; i.e., NCBI CCDS (http://www.ncbi.nlm.nih.gov/CCDS/CcdsBrowse.cgi), RefSeq (http://www.ncbi.nlm.nih.-gov/RefSeq/), Ensembl (http://www.ensembl.org), and Encode (http://genome.ucsc.edu/ENCODE). Four major steps were taken to prioritize all the high-quality variants: (i) variants within intergenic, intronic, and UTR regions and synonymous mutations were excluded from downstream analysis; (ii) variants in dbSNP137(http://www.ncbi.nlm.nih.gov/projects/SNP/), 1000 Genome project (ftp://ftp.1000genomes.ebi.ac.uk/vol1/ftp), YH Database (http://yh.genomics.org.cn/), HapMap Project (ftp://ftp.ncbi.nlm.nih.gov/hapmap) and our in-house database, which was generated by our laboratory using 1600 whole exome sequencing data, were excluded; (iii) possible damaging impacts of each variant on protein structure/function were predicted by SIFT (http://sift.bii.astar.edu.sg/) and Polyphen2 (http://genetics.bwh.harvard.edu/pph2/); (iv) gene Ontology (http://www.geneontology.org) and KEGG Pathway Annotations (http://www.ebi.ac.uk./clustalw) were used to predict the biological function of each putative gene.

### Variants Validation

After filtering against multiple databases, Sanger sequencing was used to determine whether any of the remaining variants co-segregated with the disease phenotype in this family. Primers flanking the candidate loci were designed based on genomic sequences of Human Genome (hg19/build37.1) and synthesized by Invitrogen, Shanghai, China: ABCA4-exon13-F, tgagttccgagtcaccctgt; ABCA4-exon13-R, gtcagagctccatgctctcc; ABCA4-exon16-F, ctctacctcgagggcatctg; ABCA4-exon16-R, ggctggggatctgaagaact. Genotyping for c.C2424G and c.G1819A in the family members was then confirmed by direct polymerase chain reaction (PCR) and analyzed on an ABI 3730XL Genetic Analyzer. Sequencing data were compared with the Human Genome database.

## Results

### Clinical Presentation of Family 2084

**Figure 1:**
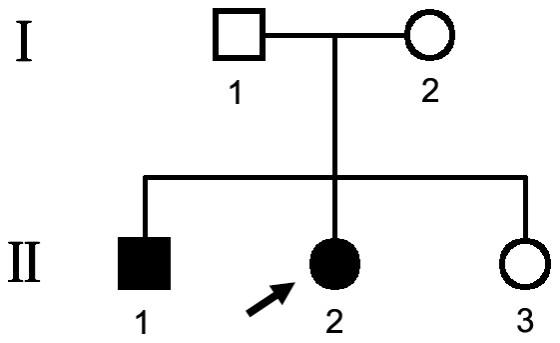
Pedigree of family 2048 with Stargardt. Solid symbols indicated affected individuals. Open symbols indicated unaffected individuals and arrow indicates the proband.

**Figure 2:**
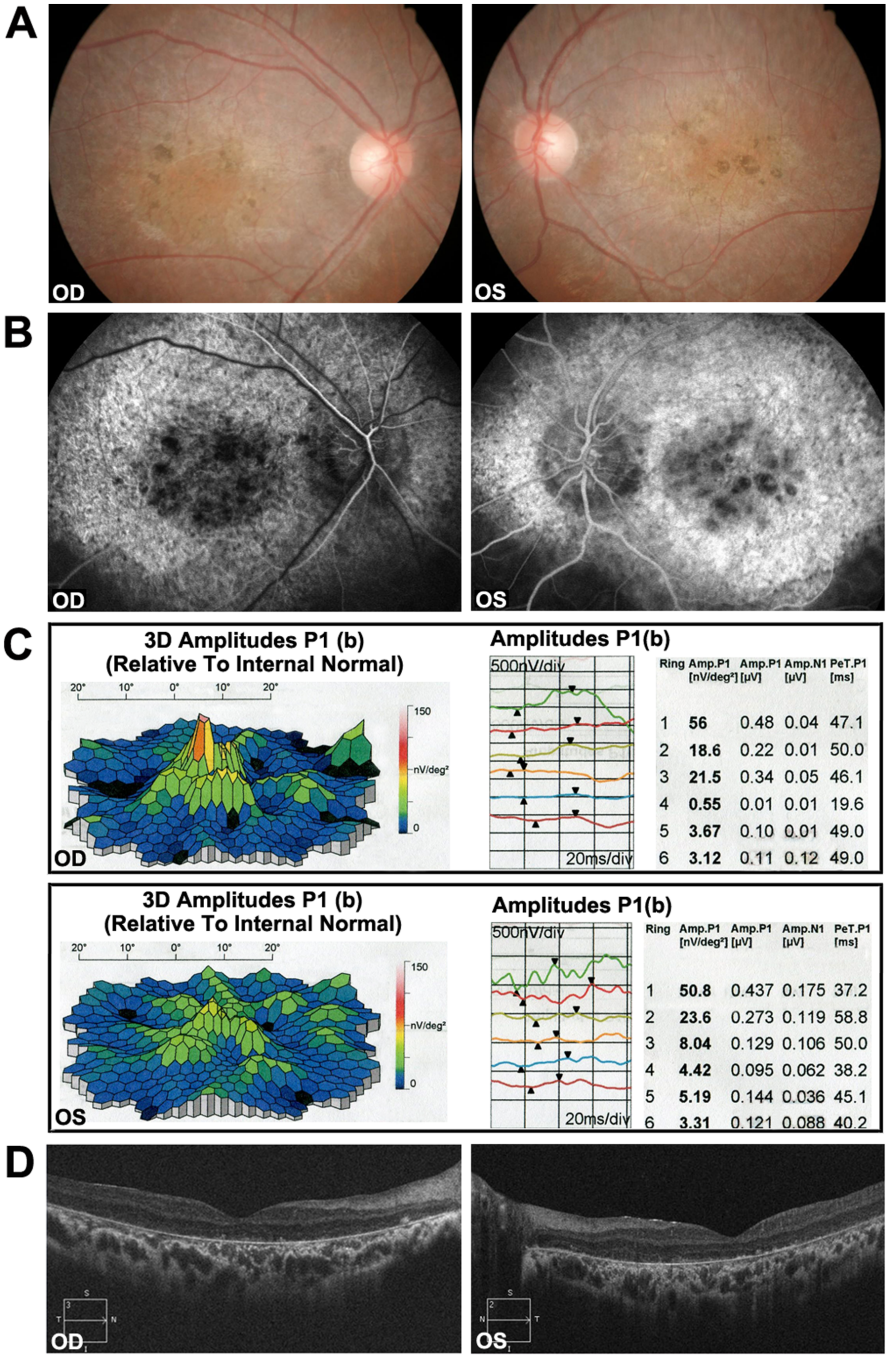
Representative photographs of patients of family 2048. (A) Fundus photographs showing pigment mottling and yellow-white flecks in both maculae. (B) Fluorescein angiography (FA) images showing the hyperfluorescent flecks extended to the midperipheral retina and fluorescence blocking by the pigment mottling in the mcular. (C) mfERG records showing severe depressed central waveform and significant paracentral/pereferral loss of retinal response. (D) Macular OCTs showing hyper-reflective deposits within the RPE layer and the level of the outer segments of the photoreceptors, thinning of the retinal outer layers and enhanced choroidal reflectivity associated with overlying atrophic retina.

A two-generation family (family 2084) from Henan Province of China was recruited in this study (Figure 1). Ophthalmic examinations identified two affected individuals as Stargardt disease patients among the five examined family members. Affected members of this family exhibited similar clinical features. The two affected siblings presented with an early-onset markedly decreased vision acuity (OD: 20/400, OS: 20/400) in both eyes and an increasing difficulty to adapt in the dark (Table 1). Fundus examination showed some pigment mottling and yellow-white flecks in both maculae, normal caliber of the retinal vessels, but no pigmented bone spicules in the retinal periphery (Figure 2A). The fluorescein angiogram displayed hyperfluorescent flecks, which extended to the midperipheral retina and fluorescence blocks formed by the pigment mottling in the macula (Figure 2B). Multifocal Electroretinogram showed severe depressed central waveform and significant loss of paracentral/peripheral retinal response (Figure 2C). The macular OCT showed hyper-reflective deposits within the RPE layer and the level of the outer segments of the photoreceptors, thinning of the retinal outer layers and enhanced choroidal reflectivity (Figure 2D).

**Table 1:** Phenotype and genotype of the family members.

Family Member	Age	Gender	Disease Duration (years)	Visual Acuity (OD, OS)	Mutation(s)		Original reports described
					Nucleotide change	Effect	
**I1**	43	M	0	20/20, 20/20	1819G>A	Gly 607 Arg	Andrea Rivera
**I2**	43	F	0	20/20, 20/20	2424C>G	Tyr 808*	N/A
**II1**	13	M	3	20/400, 20/400	1819G>A/2424C>G	Gly 607 Arg,/Tyr 808*	Andrea Rivera,N/A
**II2**	18	F	8	20/400, 20/400	1819G>A/2424C>G	Gly 607 Arg,/Tyr 808*	Andrea Rivera,N/A
**II3**	20	F	0	20/20, 20/20	1819G>A	Gly 607 Arg	Andrea Rivera

N/A, Not available; M, Male; F, Female.

### Whole-exome Sequencing

By exome sequencing of patient II:1 and patient II:2, we generated 6.9 and 7.2 billion bases of sequence with average throughput depth of target regions 111.6× and 116.4×, respectively. Approximately 99.8% and 99.8% of initial mappable reads were able to pass our thresholds for calling SNPs and short insertions or deletions (Indels). The mean read depth of target regions was 52.3× and 54.7×, respectively. In II:1 and II:2, we separately identified 20563 and 20641 SNPs in the coding regions (9377 and 9407 nonsynonymous SNPs, 10663 and 10689 synonymous SNPs, 523 and 545 other types of SNPs, respectively), 423 and 413 coding Indels that may affect amino acid sequence, respectively.

After identification of variants, we identified 5843 functional SNP and 334 functional Indels that were shared by these two patients (Table 2). We focused only on the functional SNP/Indel, including non-synonymous variants (NS), splice acceptor and donor site mutations (SS), and frameshift coding-region insertions or deletions (Indels), which were more likely to be pathogenic than others, especially those in homozygous or multiple heterozygous status. We then compared these variants in two affected members with the dbSNP135, 1000 Genome Project, HapMap project, YH database and our in-house database, which generated by our laboratory using 1600 whole exome sequencing data (table 2). The in-house data include whole exome variants data from people without any eye disease, therefore we can exclude the variants with high frequency in people without any eye disease.

**Table 2:** Number of candidate SNP/Indels filtered against several public variation databases and the in-house data.

	Feature_SNP shared by Case II1 and Case II2	Feature_Indel shared by Case II1 and Case II2
**Total_number**	49535	1174
**Functional_SNP/Indels**	5843	334
**Filtered_DBsnp135common/indel**	754	228
**Filtered_DBsnp/indel_1000gene(2011)**	753	226
**Filtered_DBsnp_1000gene_Hapmap**	752	226
**Filtered_DBsnp_1000gene_Hapmap_YH**	748	226
**Filtered in-House Data**	25	0

**Figure 3:**
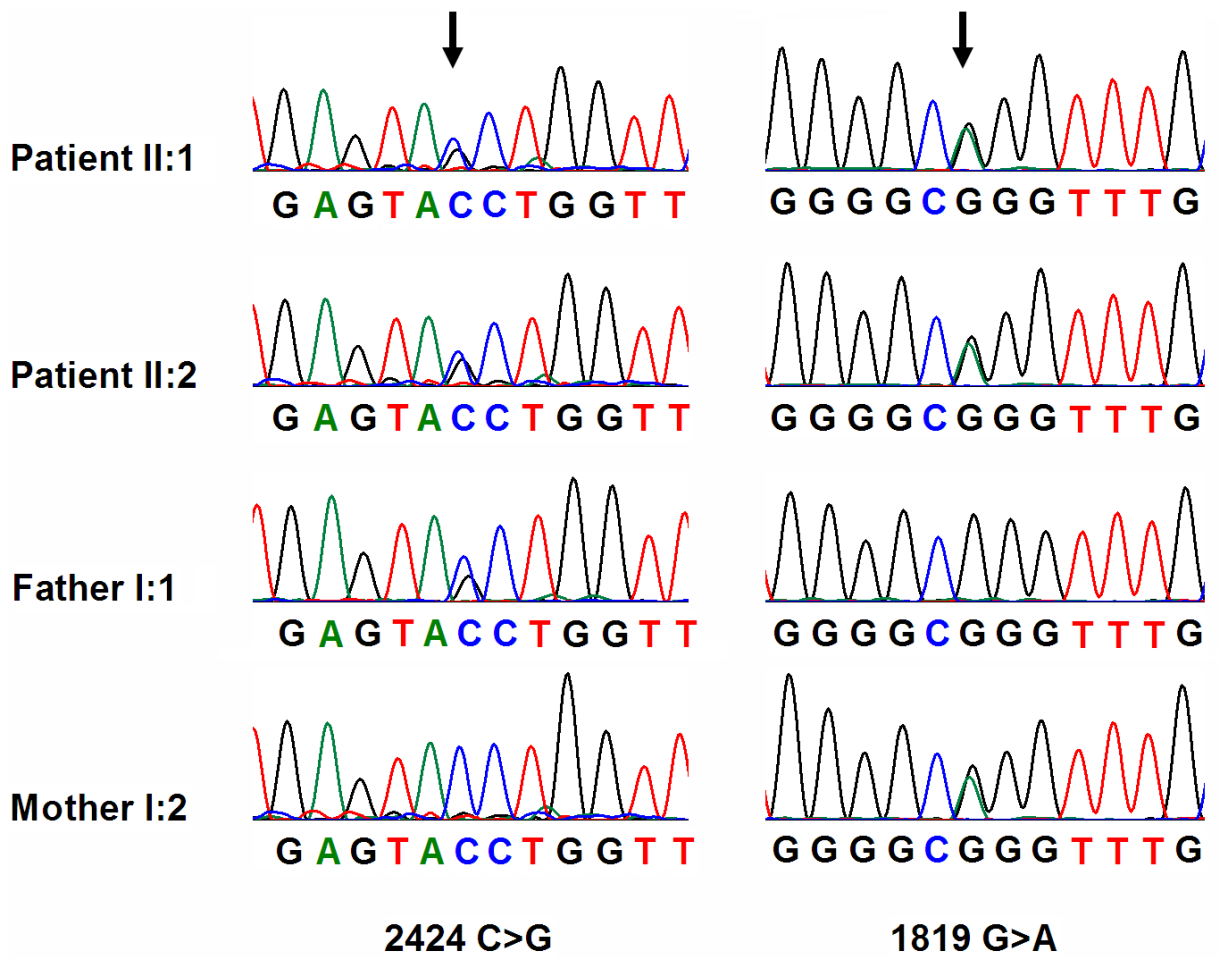
Mutation identification of *ABCA4* gene. Electropherogram analysis of *ABCA4* in family 2048 showing the compound heterozygous mutations (c.C2424G and c.G1819A) co-segregated with the phenotype. II1 and II2 patients harbored compound heterozygous c.C2424G and c.G1819A mutations of the *ABCA4* gene. c.C2424G mutation was carried by the mother I2 while c.G1819A mutation was carried by the father I1.

Under the autosomal recessive model, variants satisfying a recessive homozygous inheritance model were not identified. This led us to investigate the possibility of recessive compound heterozygous inheritance. Using this model, the filtered data was narrowed to 25 heterozygous variants. We then compared these variants with reported retina genes (https://sph.uth.edu/Retnet/). In both patients we found two mutations c.C2424G (p.Y808X) and c.G1819A (p.G607R) satisfying a recessive compound heterozygous inheritance model (Table 3) in the gene *ABCA4* (NM_000350.2). When we checked the human gene mutation database (http://www.hgmd.org/), we found that mutation p.G607R was reported by Andrea Rivera in 2000 [24]. Sanger sequencing confirmed these two mutations in the two affected siblings and demonstrated that their parents were unaffected carriers of Y808X (father) and G607R (mother) mutations, showing complete co-segregation of the mutations with the disease phenotype (Figure 3). The two mutations described above were absent in 1000 ethnicity-matched control samples screened by direct sequencing. These data, together with the clinical presentation of the two affected siblings, demonstrated that p.G607R and p.Y808X variants in the gene *ABCA4* was responsible for Stargardt disease in this family.

**Table 3:** Candidate exome sequence variants shared by all affected individuals and filtered by database.

Chr:Position	Gene	Mutation	Mode	Mutation type	dbSNP ID	Maternal Allele	Paternal Allele	SIFT/Polyphen2	Stargardt Gene
chr01:94520830	ABCA4	exon16:c.C2424G:p.Y808X	heterozygous	stopgain	Novel	Yes		Stop codon, N/A	N/A
chr01:9452825	ABCA4	exon13:c.G1819A:p.G607R	heterozygous	nonsynonymous	rs61749412		Yes	Damaging/ probably damaging	Yes

N/A, Not available.

### Mutation Detection and Analysis

SIFT was used to predict how the identified amino acid substitutions would affect protein function. The previously reported mutation p.G607R of *ABCA4* was predicted to be damaging and the novel mutation c.C2424G, p.Y808X in the affected families introduced a stop codon, which removed 1465 amino acids from the ABCA4 protein (2273 amino acids), according to GenBank accession number NM_000350.2. Therefore, this novel mutation is likely a null allele.

**Figure 4:**
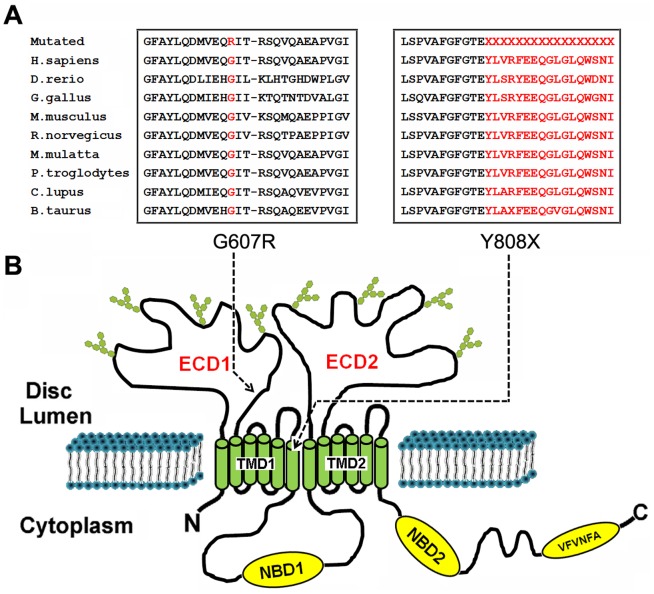
Topological organization of ABCA4 and conservation analysis. (A) Protein alignment showed conservation of residues ABCA4 Y808X and G607R across nine species. These two mutations occured at an evolutionarily conserved amino acid. The ABCA4 mutation G607R occured in the NBD1 while Y808X occured in the disc lumen region between TMD1. (B) Topological organization of ABCA4 in the disc membrane was shown. The domain organization included exocytoplamic domain (ECD), nucleotide binding domain (NBD) and transmembrane domain (TMD).

Polyphen2 was used to explore sequence homology and the physical properties of corresponding affected amino acids. Mutation p.G607R, located within exon 13, results in changes of a hydrophilic glycine to an arginine at position 607, which may lead to a damaging replacement with a score of 0.99 (sensitivity:0.72, specificity:0.97) Figure 4A. Mutation Y808X, located within exon 16, results in a nonsense mutation, and the mRNA with a premature stop codon is likely to be degenerated by the nonsense-mediated mRNA decay response, thus leading to a decrease in *ABCA4* expression. As shown in Figure 4A, both amino acid changes affect highly conserved residues.

We then used TMHMM2.0 to predict the ABCA4 protein structure. Our result showed that the protein was organized in two structurally related tandem-arranged halves with each half containing transmembrane domains (TMD) followed by nucleotide binding domains (NBD). Our result also indicated that G607R occurs in the ECD1 domain, while Y808X occurs in the disc lumen region between TMD1 (Figure 4B).

## Discussion

This study identifes novel compound heterozygous mutations in the *ABCA4* gene as a cause of Stargardt’s disease. STGD accounts for approximately 7% of all retinal dystrophies. It is one of the most common genetic forms of juvenile or early adult onset macular degeneration. This condition affects the central retina (macula) with a variable phenotype and a variable age of onset and severity.

The *ABCA4* gene, located at the chromosome 1p22.1 with 50 exons, is a large glycoprotein with 2,273 amino-acid and organized into two structurally related tandem-arranged halves with each half containing a transmembrane domain (TMD) followed by a nucleotide binding domain (NBD) [25], [26], [27]. Both the N and C halves are predicted to have a single membrane-spanning segment followed by a large exocytoplasmic (extracellular/lumen) domain (ECD), five membrane-spanning segments and a nucleotide-binding domain (NBD) [27], [28]. A highly conserved VFVNFA motif near the C-terminus has been shown to play an essential role in the folding of ABCA4 into a functional protein [29].

The ABCA4 protein is localized in cone and rod photoreceptor outer segments [30]. The normal function of ABCA4 is to facilitate the transport of all-trans-retinal from the outer segment disk to the outer segment cytoplasm in the form of a mono-substituted phospholipid known as N-retinylidene- phosphatidylethanolamine (N-ret-PE) [31], [32]. When ABCA4 is defective, N-ret-PE irreversibly forms a toxic, insoluble, bisretinoid known as A2PE which is deposited in retinal pigment epithelium (RPE) cells during the process of disc shedding and phagocytosis, eventually leading to cell death and macular degeneration [33].

Compared to wild-type mice, *Abca4* knockout mice show significant light-dependent changes in lipids and an accumulation of lipofuscin deposits in the RPE cells. Biochemical analysis of the lipofuscin deposits from *Abca4* knockout mice show elevated levels of several fluorescent diretinoid compounds, including A2E, a diretinal pyridinium compound known to be a major component of lipofuscin, all-trans retinal dimer, and related diretinal compounds [34], [35], [36], [37], [38], [39]. In addition, *Abca4* knockout mice, like individuals with Stargardt disease, show a delay in dark adaptation consistent with the delayed removal of all-trans retinal from outer segments following photobleaching [31].

Since the first report of mutations in the *ABCA4* gene by Allikmets in 1997, over 800 disease-causing mutations have been identified to date in *ABCA4*-associated phenotypes and more than half of these have been detected only once [40]. The *ABCA4* mutation spectrum includes missense, nonsense, splice-site, frame-shift, small deletion and insertion mutations, although the approximately 80% of reported changes are missense mutations. It is now generally believed that mutations in *ABCA4* result in a spectrum of related retinal dystrophies, including STGD, bull’s eye maculopathy [41], [42], retinitis pigmentosa [43], [44], [45], [46], [47], cone rod dystrophy and age-related macular degeneration [48]. Numerous genetic studies on STGD patients have revealed that the disease-associated *ABCA4* alleles are extraordinarily heterogeneous. It has been estimated that only half of STGD cases have two known or putative disease-causing *ABCA4* mutations on separate alleles, nearly one third of cases have a single mutation and the remaining have no definite or probable disease-causing *ABCA4* mutations [49].

In our study compound heterozygous mutations p.Y808X and p.G607R of the *ABCA4* gene were identified. The previously reported *ABCA4* p.G607R mutation [24], [50] in exon 13 is a single nucleotide polymorphism, rs61749412, which is predicted probably to be damaging to protein function (PolyPhen2 scores close to 1.0). Through the analysis of membrane topology by TMHMM2.0, we found that the p.G607R mutation was in the *ABCA4* ECD1 region, which is involved in stacking interactions with the adenine ring of ATP [51]. The novel stopgain p.Y808X mutation in exon 16 was detected in a heterozygous state, close to the previously reported mutation p.G818E [11]. It was predicted to result in a truncated protein, which severely impaired ABCA4 protein function. This novel compound heterozygous mutation was absent from public databases such as 1000 genomes or Exome Variant Server, excluding them as common polymorphisms.

In summary, we have reported the clinical and genetic characteristics of a Chinese family with Stargardt disease by WES. To identify pathogenic variants, we analyzed these variants by subjecting them to an analytical pipeline for high-confidence variant calling, annotation and filtration and finally identified novel compound heterozygous mutations in *ABCA4*. Our study provides another compound heterozygous mutation in *ABCA4* for Stargardt disease.

## References

[1] Weleber RG (1994) Stargardt's macular dystrophy. Arch Ophthalmol 112: 752–754. 8002831 10.1001/archopht.1994.01090180050033

[2] Walia S, Fishman GA (2009) Natural history of phenotypic changes in Stargardt macular dystrophy. Ophthalmic Genet 30: 63–68. 19373676 10.1080/13816810802695550

[3] Stargardt K (1909) Uber familiare, progressive degeenration under makulagegend des augen. Albrecht von Graefes Arch Ophthalmol 71: 534–550.

[4] Franceschetti A, Francois J (1965) [Fundus flavimaculatus]. Arch Ophtalmol Rev Gen Ophtalmol 25: 505–530. 4221555

[5] Zhang K, Kniazeva M, Han M, Li W, Yu Z, et al (2001) A 5-bp deletion in ELOVL4 is associated with two related forms of autosomal dominant macular dystrophy. Nat Genet 27: 89–93. 11138005 10.1038/83817

[6] Birnbach CD, Jarvelainen M, Possin DE, Milam AH (1994) Histopathology and immunocytochemistry of the neurosensory retina in fundus flavimaculatus. Ophthalmology 101: 1211–1219. 8035984 10.1016/s0161-6420(13)31725-4

[7] Steinmetz RL, Garner A, Maguire JI, Bird AC (1991) Histopathology of incipient fundus flavimaculatus. Ophthalmology 98: 953–956. 1866150 10.1016/s0161-6420(91)32197-3

[8] Kaplan J, Gerber S, Larget-Piet D, Rozet JM, Dollfus H, et al (1993) A gene for Stargardt's disease (fundus flavimaculatus) maps to the short arm of chromosome 1. Nat Genet 5: 308–311. 8275096 10.1038/ng1193-308

[9] Rozet JM, Gerber S, Perrault I, Camuzat A, Calvas P, et al (1996) Structure and physical mapping of DR1, a TATA-binding protein-associated phosphoprotein gene, to chromosome 1p22.1 and its exclusion in Stargardt disease (STGD). Genomics 36: 554–556. 8884286 10.1006/geno.1996.0508

[10] Nasonkin I, Illing M, Koehler MR, Schmid M, Molday RS, et al (1998) Mapping of the rod photoreceptor ABC transporter (ABCR) to 1p21-p22.1 and identification of novel mutations in Stargardt's disease. Hum Genet 102: 21–26. 9490294 10.1007/s004390050649

[11] Allikmets R, Singh N, Sun H, Shroyer NF, Hutchinson A, et al (1997) A photoreceptor cell-specific ATP-binding transporter gene (ABCR) is mutated in recessive Stargardt macular dystrophy. Nat Genet 15: 236–246. 9054934 10.1038/ng0397-236

[12] Houlston RS, Cheadle J, Dobbins SE, Tenesa A, Jones AM, et al (2010) Meta-analysis of three genome-wide association studies identifies susceptibility loci for colorectal cancer at 1q41, 3q26.2, 12q13.13 and 20q13.33. Nat Genet 42: 973–977. 20972440 10.1038/ng.670 PMC5098601

[13] Stone EM, Nichols BE, Kimura AE, Weingeist TA, Drack A, et al (1994) Clinical features of a Stargardt-like dominant progressive macular dystrophy with genetic linkage to chromosome 6q. Arch Ophthalmol 112: 765–772. 8002834 10.1001/archopht.1994.01090180063036

[14] Edwards AO, Miedziak A, Vrabec T, Verhoeven J, Acott TS, et al (1999) Autosomal dominant Stargardt-like macular dystrophy: I. Clinical characterization, longitudinal follow-up, and evidence for a common ancestry in families linked to chromosome 6q14. Am J Ophthalmol 127: 426–435. 10218695 10.1016/s0002-9394(98)00331-6

[15] Coco RM, Telleria JJ, Sanabria MR, Rodriguez-Rua E, Garcia MT (2010) PRPH2 (Peripherin/RDS) mutations associated with different macular dystrophies in a Spanish population: a new mutation. Eur J Ophthalmol 20: 724–732. 20213611 10.1177/112067211002000413

[16] Poloschek CM, Bach M, Lagreze WA, Glaus E, Lemke JR, et al (2010) ABCA4 and ROM1: implications for modification of the PRPH2-associated macular dystrophy phenotype. Invest Ophthalmol Vis Sci 51: 4253–4265. 20335603 10.1167/iovs.09-4655

[17] Petrukhin K, Koisti MJ, Bakall B, Li W, Xie G, et al (1998) Identification of the gene responsible for Best macular dystrophy. Nat Genet 19: 241–247. 9662395 10.1038/915

[18] Edwards AO, Donoso LA, Ritter R 3rd (2001) A novel gene for autosomal dominant Stargardt-like macular dystrophy with homology to the SUR4 protein family. Invest Ophthalmol Vis Sci 42: 2652–2663. 11581213

[19] Vasireddy V, Wong P, Ayyagari R (2010) Genetics and molecular pathology of Stargardt-like macular degeneration. Prog Retin Eye Res 29: 191–207. 20096366 10.1016/j.preteyeres.2010.01.001 PMC3059896

[20] Yang Z, Chen Y, Lillo C, Chien J, Yu Z, et al (2008) Mutant prominin 1 found in patients with macular degeneration disrupts photoreceptor disk morphogenesis in mice. J Clin Invest 118: 2908–2916. 18654668 10.1172/JCI35891 PMC2483685

[21] Nielsen R, Paul JS, Albrechtsen A, Song YS (2011) Genotype and SNP calling from next-generation sequencing data. Nat Rev Genet 12: 443–451. 21587300 10.1038/nrg2986 PMC3593722

[22] Ku CS, Cooper DN, Polychronakos C, Naidoo N, Wu M, et al (2012) Exome sequencing: dual role as a discovery and diagnostic tool. Ann Neurol 71: 5–14. 22275248 10.1002/ana.22647

[23] Casals F, Idaghdour Y, Hussin J, Awadalla P (2012) Next-generation sequencing approaches for genetic mapping of complex diseases. J Neuroimmunol 248: 10–22. 22285396 10.1016/j.jneuroim.2011.12.017

[24] Rivera A, White K, Stohr H, Steiner K, Hemmrich N, et al (2000) A comprehensive survey of sequence variation in the ABCA4 (ABCR) gene in Stargardt disease and age-related macular degeneration. Am J Hum Genet 67: 800–813. 10958763 10.1086/303090 PMC1287885

[25] Molday RS (2007) ATP-binding cassette transporter ABCA4: molecular properties and role in vision and macular degeneration. J Bioenerg Biomembr 39: 507–517. 17994272 10.1007/s10863-007-9118-6

[26] Illing M, Molday LL, Molday RS (1997) The 220-kDa rim protein of retinal rod outer segments is a member of the ABC transporter superfamily. J Biol Chem 272: 10303–10310. 9092582 10.1074/jbc.272.15.10303

[27] Shroyer NF, Lewis RA, Allikmets R, Singh N, Dean M, et al (1999) The rod photoreceptor ATP-binding cassette transporter gene, ABCR, and retinal disease: from monogenic to multifactorial. Vision Res 39: 2537–2544. 10396622 10.1016/s0042-6989(99)00037-1

[28] Peelman F, Labeur C, Vanloo B, Roosbeek S, Devaud C, et al (2003) Characterization of the ABCA transporter subfamily: identification of prokaryotic and eukaryotic members, phylogeny and topology. J Mol Biol 325: 259–274. 12488094 10.1016/s0022-2836(02)01105-1

[29] Zhong M, Molday LL, Molday RS (2009) Role of the C terminus of the photoreceptor ABCA4 transporter in protein folding, function, and retinal degenerative diseases. J Biol Chem 284: 3640–3649. 19056738 10.1074/jbc.M806580200 PMC4090197

[30] Weng J, Mata NL, Azarian SM, Tzekov RT, Birch DG, et al (1999) Insights into the function of Rim protein in photoreceptors and etiology of Stargardt's disease from the phenotype in abcr knockout mice. Cell 98: 13–23. 10412977 10.1016/S0092-8674(00)80602-9

[31] Quazi F, Lenevich S, Molday RS (2012) ABCA4 is an N-retinylidene-phosphatidylethanolamine and phosphatidylethanolamine importer. Nat Commun 3: 925. 22735453 10.1038/ncomms1927 PMC3871175

[32] Sheffield VC, Stone EM (2011) Genomics and the eye. N Engl J Med 364: 1932–1942. 21591945 10.1056/NEJMra1012354

[33] Mata NL, Weng J, Travis GH (2000) Biosynthesis of a major lipofuscin fluorophore in mice and humans with ABCR-mediated retinal and macular degeneration. Proc Natl Acad Sci U S A 97: 7154–7159. 10852960 10.1073/pnas.130110497 PMC16515

[34] Mata NL, Tzekov RT, Liu X, Weng J, Birch DG, et al (2001) Delayed dark-adaptation and lipofuscin accumulation in abcr+/− mice: implications for involvement of ABCR in age-related macular degeneration. Invest Ophthalmol Vis Sci 42: 1685–1690. 11431429

[35] Sparrow JR, Boulton M (2005) RPE lipofuscin and its role in retinal pathobiology. Exp Eye Res 80: 595–606. 15862166 10.1016/j.exer.2005.01.007

[36] Wu Y, Fishkin NE, Pande A, Pande J, Sparrow JR (2009) Novel lipofuscin bisretinoids prominent in human retina and in a model of recessive Stargardt disease. J Biol Chem 284: 20155–20166. 19478335 10.1074/jbc.M109.021345 PMC2740442

[37] Kim SR, Jang YP, Jockusch S, Fishkin NE, Turro NJ, et al (2007) The all-trans-retinal dimer series of lipofuscin pigments in retinal pigment epithelial cells in a recessive Stargardt disease model. Proc Natl Acad Sci U S A 104: 19273–19278. 18048333 10.1073/pnas.0708714104 PMC2148280

[38] Ben-Shabat S, Parish CA, Vollmer HR, Itagaki Y, Fishkin N, et al (2002) Biosynthetic studies of A2E, a major fluorophore of retinal pigment epithelial lipofuscin. J Biol Chem 277: 7183–7190. 11756445 10.1074/jbc.M108981200

[39] Zernant J, Schubert C, Im KM, Burke T, Brown CM, et al (2011) Analysis of the ABCA4 gene by next-generation sequencing. Invest Ophthalmol Vis Sci 52: 8479–8487. 21911583 10.1167/iovs.11-8182 PMC3208189

[40] Michaelides M, Chen LL, Brantley MA Jr, Andorf JL, Isaak EM, et al (2007) ABCA4 mutations and discordant ABCA4 alleles in patients and siblings with bull's-eye maculopathy. Br J Ophthalmol 91: 1650–1655. 18024811 10.1136/bjo.2007.118356 PMC2095527

[41] Klevering BJ, Deutman AF, Maugeri A, Cremers FP, Hoyng CB (2005) The spectrum of retinal phenotypes caused by mutations in the ABCA4 gene. Graefes Arch Clin Exp Ophthalmol 243: 90–100. 15614537 10.1007/s00417-004-1079-4

[42] Cremers FP, van de Pol DJ, van Driel M, den Hollander AI, van Haren FJ, et al (1998) Autosomal recessive retinitis pigmentosa and cone-rod dystrophy caused by splice site mutations in the Stargardt's disease gene ABCR. Hum Mol Genet 7: 355–362. 9466990 10.1093/hmg/7.3.355

[43] Birch DG, Peters AY, Locke KL, Spencer R, Megarity CF, et al (2001) Visual function in patients with cone-rod dystrophy (CRD) associated with mutations in the ABCA4(ABCR) gene. Exp Eye Res 73: 877–886. 11846518 10.1006/exer.2001.1093

[44] Fishman GA, Stone EM, Eliason DA, Taylor CM, Lindeman M, et al (2003) ABCA4 gene sequence variations in patients with autosomal recessive cone-rod dystrophy. Arch Ophthalmol 121: 851–855. 12796258 10.1001/archopht.121.6.851

[45] Klevering BJ, van Driel M, van de Pol DJ, Pinckers AJ, Cremers FP, et al (1999) Phenotypic variations in a family with retinal dystrophy as result of different mutations in the ABCR gene. Br J Ophthalmol 83: 914–918. 10413692 10.1136/bjo.83.8.914 PMC1723135

[46] Martinez-Mir A, Paloma E, Allikmets R, Ayuso C, del Rio T, et al (1998) Retinitis pigmentosa caused by a homozygous mutation in the Stargardt disease gene ABCR. Nat Genet 18: 11–12. 9425888 10.1038/ng0198-11

[47] Allikmets R, Shroyer NF, Singh N, Seddon JM, Lewis RA, et al (1997) Mutation of the Stargardt disease gene (ABCR) in age-related macular degeneration. Science 277: 1805–1807. 9295268 10.1126/science.277.5333.1805

[48] Strom SP, Gao YQ, Martinez A, Ortube C, Chen Z, et al (2012) Molecular diagnosis of putative Stargardt Disease probands by exome sequencing. BMC Med Genet 13: 67. 22863181 10.1186/1471-2350-13-67 PMC3459799

[49] Webster AR, Heon E, Lotery AJ, Vandenburgh K, Casavant TL, et al (2001) An analysis of allelic variation in the ABCA4 gene. Invest Ophthalmol Vis Sci 42: 1179–1189. 11328725

[50] Walker JE, Saraste M, Runswick MJ, Gay NJ (1982) Distantly related sequences in the alpha- and beta-subunits of ATP synthase, myosin, kinases and other ATP-requiring enzymes and a common nucleotide binding fold. EMBO J 1: 945–951. 6329717 10.1002/j.1460-2075.1982.tb01276.x PMC553140

[51] Walker JE, Saraste M, Runswick MJ, Gay NJ (1982) Distantly related sequences in the alpha- and beta-subunits of ATP synthase, myosin, kinases and other ATP-requiring enzymes and a common nucleotide binding fold. EMBO J 1: 945–951. 6329717 10.1002/j.1460-2075.1982.tb01276.x PMC553140

